# Photonic networking of quantum memories in high dimensions

**DOI:** 10.1126/sciadv.aed8404

**Published:** 2026-07-15

**Authors:** Mikhail Shalaev, Sagnik Saha, George Toh, Isabella Goetting, Ashish Kalakuntla, Harriet Bufan Shi, Jameson O’Reilly, Yichao Yu, Christopher Monroe

**Affiliations:** ^1^ Duke Quantum Center, Department of Electrical and Computer Engineering and Department of Physics, Duke University, Durham, NC 27708, USA.; ^2^ IonQ Inc., College Park, MD 20740, USA.; ^3^ Department of Physics, University of Oregon, Eugene, OR 97331, USA.

## Abstract

Quantum networking enables the exchange of quantum information between physically separated quantum systems, which has applications ranging from quantum computing to unconditionally secure communication. These quantum information is generally represented by two-level quantum systems or qubits. Here, we demonstrate a quantum network of high-dimensional (HD) quantum memories or “qudits” stored in individual atoms. The interference and detection of HD time-bin–encoded single photons emitted from atomic qudit memories herald maximally entangled Bell states across pairs of atomic qudit levels. This approach expands the quantum information capacity of a quantum network while improving the entanglement success fraction beyond the standard 50% limit of qubit-based measurement protocols.

## Teaser

High-dimensional entanglement between remote quantum memories enables more efficient and scalable quantum networking.

## INTRODUCTION

Scalable quantum computing will likely require optical photonic interconnects for modular and replicable architectures, regardless of the underlying physical platform. In such architectures, quantum memories store and process quantum information, while single photons distribute quantum information across the network (*1*, *2*). While most quantum information processing relies on two-level systems or qubits, many quantum platforms naturally have multilevel structures that can serve as high-dimensional (HD) quantum memories. Qudits are quantum memories with d distinct levels, and n qudits offer access to a $d^{n}$-dimensional Hilbert space. Qudits promise more efficient resource utilization, enable a richer set of algorithms for quantum computing and simulations (*3*–*6*), and support advanced protocols for quantum networking (*7*–*9*) and quantum key distribution (*10*–*12*).

Many quantum platforms support qudit memories. Superconducting Josephson junctions have natural qudits based on the anharmonicity of their oscillators (*13*, *14*), although it remains a great challenge to couple them to optical photons with high fidelity (*15*). Entanglement between atomic ensemble qudits has been explored (*16*), although such qudits cannot readily be directly manipulated or entangled with other qudit memories. HD single photons have been realized using various degrees of freedom such as time, frequency, and spatial mode (*7*), and two photons have been entangled in a (100 × 100)–dimensional Hilbert space (*17*). Atoms offer access to multiple long-lived metastable states suitable for qudit encoding (*3*–*6*, *18*–*20*) and are a natural interface to pure single photons (*21*) enabling quantum networking (*22*–*25*). Moreover, atoms are perfectly replicable quantum memories that can exhibit excellent coherence (*26*), nearly perfect state preparation and measurement (SPAM) (*27*), and high-performance local entangling gate operations (*28*, *29*).

Here, we generate HD time-bin–encoded single photons entangled with single atomic qudit memories, up to d=4 dimensions. With two such physically separated atom-photon qudit systems, we probabilistically generate Bell-like remote entanglement between the two atomic qudit memories by interfering and detecting the two HD photons. We demonstrate qudit entanglement fidelities up to 0.987(13). Last, we directly verify the expected photonic qudit heralding success fraction F=1−1/d, exceeding the standard Bell-state generation limit of ^1^/_2_ for qubits (d=2).

## RESULTS

### Qudit memory-photon interface

**Fig. 1.**
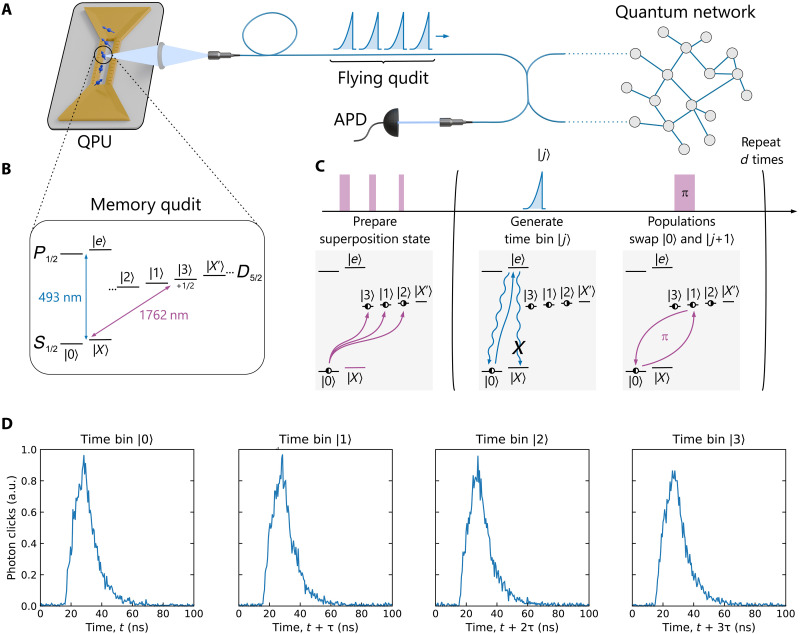
Single atom-photon interface. (**A**) A qudit-based quantum processing unit (QPU) containing quantum memories stored in multiple electronic states of trapped atomic ions. The populations stored in these levels are entangled with HD time-bin–encoded single photons, which serve as flying qudits for quantum networking applications. (**B**) Relevant energy levels of a ^138^Ba^+^ atomic ion. The qudit basis states ∣0〉, ∣1〉, ∣2〉, and ∣3〉 are encoded in the ${}^{2}S_{1/2}$ and ${}^{2}D_{5/2}$ manifolds and manipulated using a narrow-band 1762-nm laser. Lasers operating at 493 and 650 nm are used for Doppler cooling, initialization, and readout. (**C**) Atom-photon entanglement protocol. The atomic qudit is first prepared in a superposition of all its states. A pulsed 493-nm laser excites ∣0〉 to the short-lived excited state ∣e〉, generating the first time bin of a single photon correlated with state ∣0〉. Next, the qudit populations are swapped with additional 1762-nm pulses, and the excitation step is repeated to generate the other time bins. (**D**) Arrival times of 6106 photons detected in four time bins separated by τ=5680 ns, measured with an avalanche photodetector (APD). Each peak follows a 7.86-ns exponential decay, broadened by electronic and detector response delays. a.u., arbitrary units.

We begin by generating single photons encoded in up to four distinguishable time windows, or time bins, emitted from a single atom (Fig. 1A). Specific atomic levels are coherently correlated with the corresponding time bins of a single photon, enabling entanglement between atom and photon. We encode the qudit state in up to four ground and metastable levels of a single ^138^Ba^+^ atomic ion defined by ${|0\rangle \equiv |}^{2}S_{1/2},m_{J}=-\frac{1}{2}\rangle$ and ${|\left\{1,2,3\right\}\rangle \equiv |}^{2}D_{5/2},m_{J}=\left\{-\frac{1}{2},-\frac{3}{2},+\frac{1}{2}\right\}\rangle$, as shown in Fig. 1B. For the optical transitions between ∣0〉 and the metastable levels, the laser polarization is aligned to ensure nearly equal Rabi frequencies, and the spectrally resolved transitions are selectively driven by precise frequency tuning of the excitation laser. We also include ground and metastable error states ${|X\rangle \equiv |}^{2}S_{1/2},m_{J}=+\frac{1}{2}\rangle$ and ${|X^{\prime }\rangle \equiv |}^{2}D_{5/2},m_{J}=+\frac{3}{2}\rangle$, which are used to veto infrequent erasure errors during photon generation described below (*23*).

Figure 1C shows the experimental protocol and pulse sequence for generating entanglement between the atom’s internal levels and a single photon’s time-bin degree of freedom. The protocol begins by Doppler cooling the atomic ion using 493- and 650-nm light. The atom is optically pumped to the ∣0〉 state and initialized in an equal superposition state $|\psi_{0}\rangle =\frac{1}{\sqrt{d}}\sum_{j=0}^{d-1}|j\rangle$ by sequentially driving transitions to the metastable states using 1762-nm laser pulses with areas $2\;\text{arcsin}\;\frac{1}{\sqrt{d+1-n_{\text{pulse}}}}$, where $n_{\text{pulse}}=\left\{1,\ldots ,d-1\right\}$. Next, a 3-ps $\sigma^{+}$-polarized 493-nm optical pulse excites population from ∣0〉 to the state ${|e\rangle \equiv |}^{2}P_{1/2},m_{J}=+\frac{1}{2}\rangle$, which then spontaneously decays back to ∣0〉, emitting an exponentially decaying wave packet with a characteristic lifetime of 7.86 ns, thus generating the first time bin of a single photon. Additional time bins are generated by subsequently swapping the population between ∣0〉 and ∣j〉 states, followed by an excitation pulse as before (*30*). The time-bin separation is made synchronous with the atomic ion’s motional modes to avoid entanglement with atomic motion, which could otherwise degrade photon coherence (*23*, *31*–*33*). Figure 1D displays the single-photon detection timing histogram on an avalanche photodetector (APD) over four time bins with a spacing of τ=5680 ns between pulsed excitations.

We collect the emitted photon with a high–numerical-aperture objective and couple into a single-mode fiber. Conditioned on successful photon collection, the atom-photon system is ideally left in a maximally entangled state

$${|\psi \rangle }_{\text{atom}‐\text{photon}}\;=\frac{1}{\sqrt{d}}\sum_{j=0}^{d-1}{|j\rangle }^{\left(a\right)}{|j\rangle }^{\left(a\right)}{|j\rangle }^{\left(p\right)}$$ (1)

where ${|j\rangle }^{\left(p\right)}$ denotes a photon present in the $j^{\text{th}}$ time bin at the fiber’s input and ${|j\rangle }^{\left(a\right)}$ refers to the atom’s internal qudit state. For clarity, we omit the increment of atom state from ∣j〉 to ∣j+1〉 due to population swapping during time-bin generation. These HD single photons have potential applications in quantum communication protocols and can, in principle, mediate HD entanglement with a second, spatially separated qudit memory, establishing the foundation for networking multiple qudit-based quantum processing units (QPUs), as depicted in Fig. 1A.

In the experiment, we generate two such atom-photon qudit entangled states from the two separate systems labeled A and B. The end-to-end photon collection and detection probabilities for each system are $p_{A}=0.005$ and $p_{B}=0.007$. Each probability is a product of the solid angle of light collection, optical losses (including fiber coupling and beam splitter loss), and the detector efficiency, as detailed in the Supplementary Materials. On the basis of downstream measurements of entanglement between two qudit atomic memories as described below, we infer a bound (*34*) on the atom-photon entanglement fidelities of each atom-photon system to be F>{0.959,0.931,0.913} for qudit dimensions d={2,3,4}.

### Networking qudit memories

When two separate atom-photon HD entangled states are generated as outlined above, the atomic qudits can be entangled through Bell-state measurements (BSM) of the two HD photons (*35*). BSM is a cornerstone of quantum networking and essential for quantum repeater operation and establishing long-distance quantum communication links (*35*, *36*). These measurements rely on two-photon interference, which erases which-path information and projects remote memories into entangled states.

**Fig. 2.**
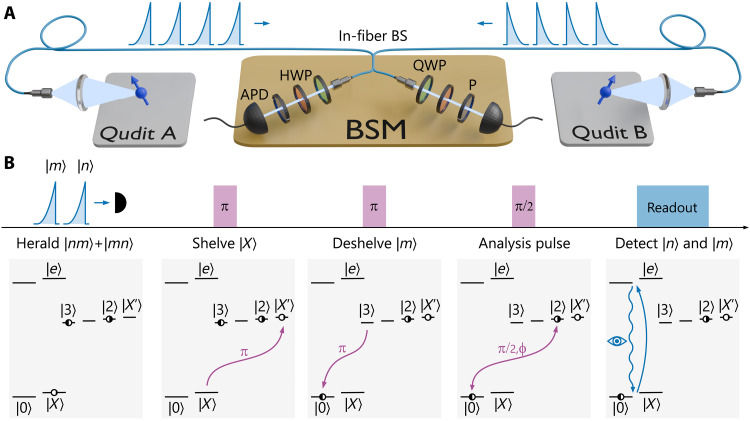
Two-qudit network. (**A**) Schematic of the experimental setup with two vacuum chambers labeled qudit A and B, each containing a single trapped ^138^Ba^+^ ion. Each atom is initialized in a qudit superposition state and emits a time-bin–encoded single photon entangled with its internal electronic state. The two photons interfere at a beam splitter (BS) and, after passing through polarizers (Ps), are detected by APDs. Quarter- and half-wave plates (QWPs and HWPs) are used to compensate polarization rotation in the optical fiber. Coincident detection of two photons in time bins n and m ideally heralds entangled atom-atom states of the form ${|n\rangle }_{A}{|m\rangle }_{B}\pm {|m\rangle }_{A}{|n\rangle }_{B}$, where n,m∈{0,1,2,3} and m>n. (**B**) Entanglement generation protocol. Following atomic excitation and successful heralding of two photons shown in (A) detected in time bins ∣n〉 and ∣m〉, each atomic qudit is measured. First, residual population in the state ∣X〉 is shelved to the metastable $|X^{\prime }\rangle$ state, and then a series of shelving pulses sequentially transfers the expected qudit state to the ∣0〉 state for fluorescence detection. If there is no fluorescence in either state ∣n〉 or ∣m〉 for either atom, then the event is discarded. Example is shown for entangled states involving n=2 and m=3.

In the experiment, we entangle two qudit memories of dimensions d={2,3,4} residing in separate ion trap systems positioned ∼2 m apart. Figure 2 shows the experimental apparatus, the entanglement generation protocol, and the corresponding control sequence (see the Supplementary Materials). We follow the same procedure as in the previous section, now exciting the two atomic qudits with synchronized short laser pulses and generating single photons from each atom. The photons are then spatially mode matched on a beam splitter and detected on the output ports. Detection of two photons in time bins n and m (m>n) heralds the creation of a maximally entangled Bell state between the two memories, ideally given by

$$|\Psi^{\pm }\rangle =\frac{{|n\rangle }_{A}{|m\rangle }_{B}\pm e^{\mathrm{i\phi}}{|m\rangle }_{A}{|n\rangle }_{B}}{\sqrt{2}}$$ (2)

The sign of the entangled state above is “+” when the photons are detected on the same side of the beam splitter and “–” when they are detected on opposite sides. The optical phases acquired when propagating from the atoms to the beam splitter cancel in the above state, as the optical path is stable between time bins n and m. The residual phase ϕ≪1 in Eq. 2 accounts for known relative level splittings between the two qudits, described below and in Materials and Methods.

Once a BSM is performed and two detection events are registered in time bins n and m, the atomic qudit states are measured via standard fluorescence detection after coherently transferring populations from the ∣n〉 and ∣m〉 states of each atom to their $|{}^{2}S_{1/2}\rangle$ ground state manifolds (*5*, *37*). The atomic qudit state detection fidelities are better than 0.99, including imperfections in the population transfers.

**Fig. 3.**
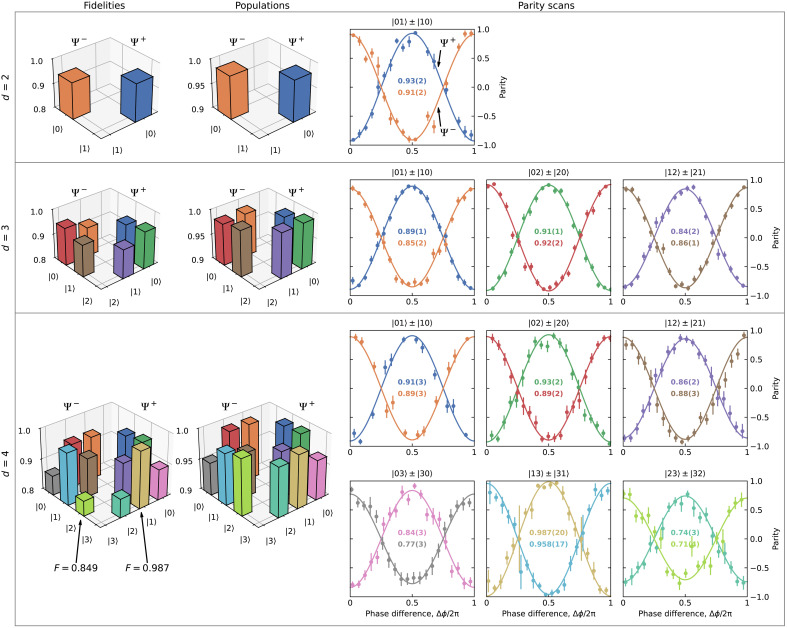
Qudit entangled state characterization. The measured fidelity of each entangled qudit state $\Psi^{\pm }={|n\rangle }_{A}{|m\rangle }_{B}\pm {|m\rangle }_{A}{|n\rangle }_{B}$ for dimensions d=2, d=3, and d=4 is displayed on the left column for each value of n and m. The fidelities are calculated from the population measurements (middle column) and the contrast of the parity scans (right column). The parity oscillations are measured by inserting global π/2 rotations with phase difference Δϕ after heralding but before measurement, with statistical error bars and best-fit contrasts indicated in each plot. All data include SPAM errors.

The fidelity of the entangled state with respect to an ideal Bell state is given by F=(P+C)/2, where P is the probability of occupying states ${|n\rangle }_{A}{|m\rangle }_{B}$ or ${|m\rangle }_{A}{|n\rangle }_{B}$ following a measurement and C/2 is the magnitude of the off-diagonal coherence between the two states (*38*). We measure the coherence by applying π/2 rotations with relative phase Δϕ to the atoms before fluorescence detection. The value of C is the contrast of the parity oscillations of the two atomic states as Δϕ is scanned, and the states with opposite signs in Eq. 2 are shifted by π phase. The measured populations and parity scans for all entangled states from Eq. 2 are displayed in Fig. 3. We observe entangled state fidelities in a range between 0.849(23) and 0.987(13) (not adjusted for SPAM errors).

We now discuss the sources of observed fidelity imperfections. Differential magnetic field drift between the two qudit systems will affect the phase of the entangled states in Eq. 2. We mitigate this phase drift error under the assumption that the magnetic field drift varies over a time scale much slower than the entanglement generation rate. This allows us to monitor the phase for each heralded state in Eq. 2 and keep track of its slow drift given the known sensitivity of each qudit state to magnetic fields. During data analysis, we feed-forward the phase accumulation to recover contrast degradation from slow magnetic field noise, as described in Materials and Methods.

When the photons are produced, undesired decay events to the ∣X〉 level (Fig. 1C) are largely suppressed through polarization filtering. Residual events are converted into erasure errors by shelving the ∣X〉 state to the metastable $|X^{\prime }\rangle$ state (Fig. 2B), thereby rendering the atom dark under fluorescence detection, as detailed in Materials and Methods. If either atom appears dark during state detection, the trial is discarded. We rejected between 6 to 11% of data depending on the qudit dimensionality, allowing for similar fidelity improvements (*23*).

Increased dimension requires more population swapping operations, increasing overall error for certain entangled Bell states. The observed errors are primarily due to decoherence from fast differential magnetic field fluctuations and state swapping imperfections. The measured fidelities of the various Bell states differ because of their unique sensitivities to magnetic field noise and contributions from state swapping errors, as detailed in the error budget in the Supplementary Materials. Some of these errors can be mitigated by hyperfine states, which are insensitive to magnetic field drifts to the first order, and by stabilizing the power and pointing noise of the population-swapping laser. Nonetheless, several Bell states exhibit fidelities consistent with prior two-qubit entanglement benchmarks (*23*).

### Qudit Bell state success fraction

With every attempt, the probability of successfully generating atom-atom entanglement is $P_{\text{ent}}=Fp_{A}p_{B}$, where F is the qudit heralding success fraction. The success probability of BSM for two qubits (d=2) using passive linear optics is fundamentally limited to F=1/2 because two of the four Bell states produce indistinguishable coincidence detection patterns (*39*, *40*). This limitation can be overcome using additional photonic qubits (*41*, *42*), entanglement across multiple degrees of freedom (*43*, *44*), nonlinear interactions at the single-photon level (*45*), or complex feed-forward control (*46*, *47*). Alternatively, HD single photons enable higher BSM success probabilities without additional complexity.

For two qudits each prepared in an equal superposition of its d states and correlated with its photon qudit as in Eq. 1, there are $d^{2}$ possible input states to the beam splitter. Of these, d states produce photons in the same time bin (n=m), which do not produce entanglement and are discarded. After heralding and detection, this leaves $d^{2}-d$ entangled states of the qudits in the form of Eq. 2. The qudit entanglement success fraction is therefore F=1−1/d (*8*, *35*).

**Fig. 4.**
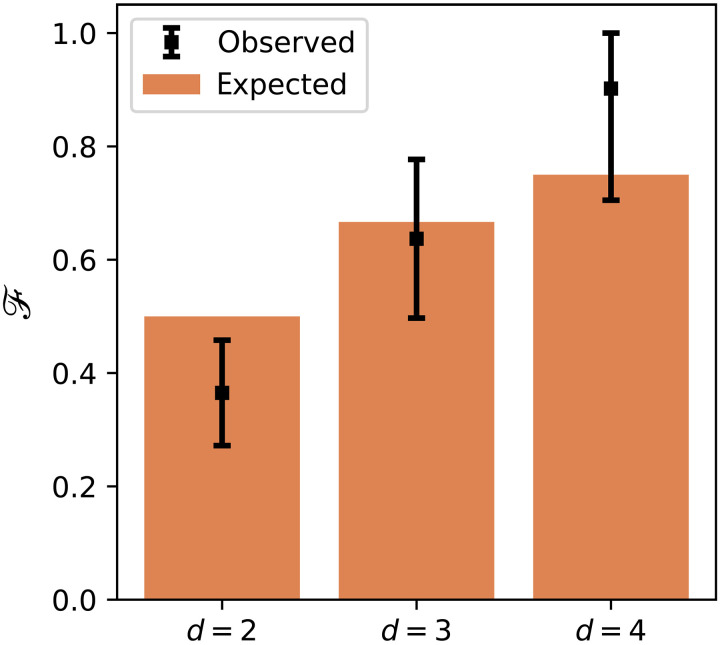
Bell-state measurement success fraction. Measured BSM success fractions for qudit dimensions d={2,3,4} (black points and statistical error bars). The success fraction increases with dimension according to the theoretical scaling F=1−1/d (orange bars), which reflects the fraction of distinguishable antisymmetric Bell states.

Figure 4 displays the observed and ideal qudit heralding success probabilities for dimensions d={2,3,4}. The success fractions F were extracted from the experimental atom-atom entanglement success probabilities $P_{\text{ent}}$ by factoring out the known efficiencies $p_{A}$ and $p_{B}$ for each atom-photon system (see the Supplementary Materials and table S2). The observed success fraction increases with dimension as expected, overcoming the fundamental Bell-state detection limit.

## DISCUSSION

Qudit memories and HD photons can substantially expand the capacity of quantum information networks and offer key advantages for advancing both quantum computing and quantum communication. While the form of entanglement generated in this work is limited to Bell states stored in qudits, this can be expanded to HD entanglement spanning every level of the qudit manifold. For example, HD entanglement between two qudit memories can be established via a single HD photon using pitch-and-catch schemes: One memory emits the photon, which is then absorbed by a second memory, directly transferring the quantum state (*48*–*50*). Distributing such HD entanglement across remote quantum memories enables qudit teleportation (*51*) and the implementation of generalized qudit control gates between network nodes (*52*), thereby supporting distributed quantum computation with qudit memories (*53*). Leveraging HD memories together with native qudit gates provides a resource-efficient approach to quantum information processing and may play a key role in scalable quantum network architectures (*3*, *52*).

## MATERIALS AND METHODS

### Mitigation of slow magnetic field drifts through feed forward

The entangled states of Eq. 2 are insensitive to common-mode magnetic field noise due to their symmetry. However, differential magnetic field noise between the qudits will affect the phase of their coherence relative to an outside local oscillator (or other quantum systems to be networked). We can absorb that phase by writing the effective entangled state as

$$|\psi \left(t^{\prime }\right)\rangle =\frac{{|n\rangle }_{A}{|m\rangle }_{B}\pm e^{-\mathrm{i\phi}}{|m\rangle }_{A}{|n\rangle }_{B}}{\sqrt{2}}$$ (3)

Here $\phi =\delta {\mathrm{B\gamma}}_{\mathrm{nm}}T$, where δB is the average magnetic field difference between the two qudit positions A and B over time interval T between the heralding detection and the final analysis π/2 pulse. The magnetic field sensitivity $\gamma_{\mathrm{nm}}$ depends on the relative Zeeman shifts of the particular atomic levels ∣n〉 and ∣m〉 involved, given by their relative magnetic *g*-factors. In addition, the atoms are shelved and swapped through various field-sensitive states depending on the dimension d of the final entangled state, resulting in a complicated overall sensitivity of each entangled state in Eq. 3 to the magnetic field.

During data analysis, we make fine corrections to the phase of each coherence measurement in the parity scan through a feed-forward technique that effectively compensates for a slowly varying differential magnetic field. We determine the particular differential magnetic field δB at each time that minimizes the deviation of each adjusted phase from that expected by the known phase sensitivity.This feed-forward adjustment effectively rephases the entangled states and improves the measured state coherence (parity scan contrast) by up to ≈22% for the most sensitive state. Further details on the fitted magnetic-field drifts and the corresponding phase shifts of the different Bell states are provided in the Supplementary Materials.

### Fidelity of atom-photon state

The BSM experiments only measure the coherence between each pairs of the atomic qudit states and are not a direct measurement of the HD atom-photon entangled states. However, the results of the BSM provide constraints to the density matrix of the atom-photon state ρ atom-photon and can therefore be used to compute a lower bound of its fidelity.

The atom-atom population results P provide lower bounds of the probability for the atom-photon states to be in the correlated subspace

$$\left\{\left.{|j\rangle }^{\left(a\right)}{|j\rangle }^{\left(p\right)}\right|\;0⩽j<d\right\}$$ (4)

This reduces the density matrix to only the elements

$${\rho_{\mathrm{ij}}\equiv \langle i|}^{\left(a\right)}{\langle {i|}^{\left(p\right)}\rho_{\;\text{atom}‐\text{photon}}|j\rangle }^{\left(a\right)}{|j\rangle }^{\left(p\right)}$$ (5)

The ratio for a particular pair of atom-atom Bell states to be detected $r_{\mathrm{ij}}$ constrains the diagonal density matrix elements from the atom-photon density matrix $\rho_{\mathrm{ii}}$

$$r_{\mathrm{ij}}=\rho_{\mathrm{ii}}^{A}\rho_{\mathrm{jj}}^{B}+\rho_{\mathrm{jj}}^{A}\rho_{\mathrm{ii}}^{B}$$ (6)

where the A and B superscripts on the density matrix elements denote the two ions. The parity contrast $C_{\mathrm{ij}}$ of an atom-atom Bell states is proportional to the product of the off-diagonal density matrix elements from the two atom-photon pairs

$$C_{\mathrm{ij}}=\frac{|\rho_{\mathrm{ij}}^{A}\rho_{\mathrm{ji}}^{B}|}{r_{\mathrm{ij}}}$$ (7)

However, because the phase of the BSM parity scan is the sum of that for the two atom-photon states and does not constraint the atom-photon phase individually, we conservatively allow the phase for the $\rho_{\mathrm{ij}}$ to vary freely and only constrain it by the legality of the density matrix itself, i.e., that $\rho_{\mathrm{ij}}$ must be positive semidefinite. Combining these constraints, we numerically optimized the density matrix element $\rho_{\mathrm{ij}}$ to find the lowest atom-photon fidelity that is consistent with our BSM results.

### Erasure conversion

When single photons are emitted via spontaneous decay, polarization errors can arise because of inhomogeneous birefringence of optical components (such as vacuum windows), which can mix $\sigma^{+}$ and π polarizations and lead to false-positive heralding events. These errors are accompanied by ion population decaying to the ∣X〉 state and are mitigated by driving this population to the $|X^{\prime }\rangle$ level and rendering the ion dark during both our state detections (for n and m states), thus converting false heralds into erasure errors as described in (*23*). In our protocol, we postselect on events in which both ions are detected in the n or m states via sequential state-selective fluorescence and discard any events in which an ion is detected as dark in either measurement. We reject {11.5,6.4,9.1}% of data for qudit dimensions d={2,3,4}, respectively. Discarding events in which one or both atoms are outside the intended state subspace allows to similar improvements in the measured state fidelities.

## Supplementary Materials

### This PDF file includes:

Supplementary Text Figs. S1 to S4 Tables S1 to S5 References
