# Supplementary material for: Photonic networking of quantum memories in high dimensions
Source: Sci Adv. 2026 Jul 15;12(29):eaed8404. doi: 10.1126/sciadv.aed8404 (PMC13371916; doi:10.1126/sciadv.aed8404)
Supplement: Supplementary file 1 — Supplementary Text Figs. S1 to S4 Tables S1 to S5 References [file sciadv.aed8404_sm.pdf]

Science Advances
AAAS

Supplementary Materials for

Photonic networking of quantum memories in high dimensions

Mikhail Shalaev *et al.*

Corresponding author: Mikhail Shalaev, mikhail.shalaev@duke.edu

Sci. Adv. **12**, eaed8404 (2026)
DOI: 10.1126/sciadv.aed8404

This PDF file includes:

Supplementary Text
Figs. S1 to S4
Tables S1 to S5
References

A The entangling protocol

The ions are first Doppler-cooled and optically pumped into the ground state $|0\rangle$. Each ion is then prepared in an equal superposition so the resulting two-ion state can be written as:

$$|\psi_0\rangle = \frac{1}{d}\sum_{j=0}^{d-1}|j\rangle_A^{(a)} \otimes \sum_{q=0}^{d-1}|q\rangle_B^{(a)} \tag{S1}$$

where d is the qudit dimension. The superscript (a) denotes an atomic ion state, and the labels $\{A, B\}$ refer to the ions in the A and B chambers, respectively.

To generate a photon in a specific time-bin, the ion population in $|0\rangle$ is promoted to the excited state $|e\rangle$, from which it may spontaneously decay back to $|0\rangle$, emitting a single photon in time-bin j. The operator for such a process is:

$$A_j = |j\rangle^{(a)}\langle j|^{(a)}\left(\sqrt{p}e^{i\mathbf{\Delta k}\cdot\mathbf{r}_{A,B}(t_j)}c_j^\dagger + \sqrt{1-p}\right) + \sum_{q\neq j}^{d-1}|q\rangle^{(a)}\langle q|^{(a)} \tag{S2}$$

where p is the overall probability to detect the photon, $c_j^\dagger$ is the photon creation operator for time-bin j, $\mathbf{\Delta k} = \mathbf{k'} - \mathbf{k}$ is the difference between the excitation pulse wavevector $\mathbf{k'}$ and the emitted photon wavevector $\mathbf{k}$; and $\mathbf{r}_{A,B}(t_j)$ is the atomic position operator for ion A or B.

The two ion-photon joint states at the beamsplitter input, conditioned on successful photon emission in time-bin n and collection into an optical fiber, is given by:

$$|\psi_1\rangle = |n\rangle_A^{(a)}\sum_{j>n}^{d-1}|j\rangle_B^{(a)}|n\rangle_A^{(p)}e^{i\phi_{A,n}} + |n\rangle_B^{(a)}\sum_{j>n}^{d-1}|j\rangle_A^{(a)}|n\rangle_B^{(p)}e^{i\phi_{B,n}} \tag{S3}$$

where $\phi_{\{A,B\},n} = \mathbf{\Delta k}\cdot\mathbf{r}_{\{A,B\}}(t_n) + \phi^{\text{opt}}_{\{A,B\}}$ accounts for the optical and motional phases; $\mathbf{r}_{\{A,B\}}(t_e)$ is the position of an ion $\{A, B\}$ at the time t_n; the superscript (p) denotes photonic modes, and $\{A, B\}$ subscripts indicates the presence of a photon in the input modes A or B of a beamsplitter.

The photons are interfered on a beamsplitter, so we apply the beamsplitter transformation $|n\rangle_{\{A,B\}} \longrightarrow |n\rangle_C \pm |n\rangle_D$, yielding the atom-atom state conditioned on a detection in output modes C or D:

$$\begin{aligned}|\psi_2\rangle =&|n\rangle_C^{(p)}\left(|n\rangle_A^{(a)}\sum_{j>n}^{d-1}|j\rangle_B^{(a)}e^{i\phi_{A,n}} + |n\rangle_B^{(a)}\sum_{j>n}^{d-1}|j\rangle_A^{(a)}e^{i\phi_{B,n}}\right)\\ &+|n\rangle_D^{(p)}\left(|n\rangle_A^{(a)}\sum_{j>n}^{d-1}|j\rangle_B^{(a)}e^{i\phi_{A,n}} - |n\rangle_B^{(a)}\sum_{j>n}^{d-1}|j\rangle_A^{(a)}e^{i\phi_{B,n}}\right).\end{aligned} \tag{S4}$$

To generate the other time-bins, we swap the population between $|0\rangle$ and $|j\rangle$ and apply an excitation pulse. Upon successful detection of a second photon in time-bin $m > n$, the entangled atom-atom state becomes:

$$|\psi_3\rangle = \left(|n\rangle_C^{(p)}|m\rangle_C^{(p)} - |n\rangle_D^{(p)}|m\rangle_D^{(p)}\right)\left(|n\rangle_A^{(a)}|m\rangle_B^{(a)}e^{i\Delta\phi} + |m\rangle_A^{(a)}|n\rangle_B^{(a)}\right)$$
$$+\left(|n\rangle_C^{(p)}|m\rangle_D^{(p)} - |n\rangle_D^{(p)}|m\rangle_C^{(p)}\right)\left(|n\rangle_A^{(a)}|m\rangle_B^{(a)}e^{i\Delta\phi} - |m\rangle_A^{(a)}|n\rangle_B^{(a)}\right) \quad \text{(S5)}$$

where $\Delta\phi = \left(\phi_{A,n} - \phi_{A,m}\right) - \left(\phi_{B,n} - \phi_{B,m}\right)$ is differential phase for time-bins n and m.

In our implementation, we assume synchronization between pulsed excitations and ion motion periods, and negligible optical phase drift between time-bin generations. Under this assumption, optical and motional phase contributions cancel for time-bins n and m, reducing the overall phase to a global phase that can be neglected.

Note that in our experiments, the final atomic states are different due to the swap pulses used for time-bin generation. This advances the state as $\{n, m\} \rightarrow \{n+1, m+1\}$ (if $m = d$, then $m \rightarrow 0$).

B Remote entanglement rate and success probability

Fig. S1 presents the remote qudit-qudit entanglement generation rates and entanglement generation success probabilities measured across multiple data sets for each qudit with dimension d. Each data point corresponds to a separate acquisition run. Dashed lines indicate the average rate for each value of d.

The remote entanglement rate is written as

$$R = \frac{\mathscr{F} p_A p_B}{\tau_0 + \tau_{\text{bin}}(d-1)}, \quad \text{(S6)}$$

where τ_0 is the entanglement attempt time excluding time-bin generation and τ_{bin} is the overhead for the generation of extra time-bins. $\mathscr{F} = 1 - 1/d$ is the dimension-dependent entanglement success fraction. The overhead for adding extra dimensions to qudits may outweigh the gains in the entanglement rate, so the optimal trade-off may depend upon how quickly the extra dimensions (in our case time-bins) can be generated.

The entanglement rate is primarily limited by the duration of single-qubit operations, which depends on the available optical power and the optics used for beam focusing. A further limitation arises from the need to synchronize time-bin generation with the ion's motional period. This constraint may be relaxed by increasing trap frequencies so that the time-bin delay is much shorter than the period of atomic motion (*54*), or by cooling ions to near the motional ground state, perhaps through continuous sympathetic cooling (*37*).

The entanglement rates vary with qudit dimension due to the increasing overhead required to generate and manipulate additional time-bins. For $d = \{2, 3, 4\}$, the entanglement cycle periods are $\tau_{\text{cycle}} \approx \{17.0, 22.7, 34.1\}$ μs, respectively. These durations also depend on the Rabi frequency of the 1762 nm single-qubit rotations, which are regularly re-calibrated during data collection to ensure high-fidelity rotations. Table S1 lists other relevant metrics from our experiment for each qudit dimension, including the number of successes and attempts.

The atom-atom entanglement success probability is given by $P_{\text{ent}} = \mathscr{F} p_A p_B$, as defined in the main text. The reported values of P_{ent} are directly calculated from the total number of successes and attempts during our experiment. The probability of light collection and detection from each chamber denoted p_A and p_B are products of various efficiencies and losses as listed in Table S2. To

extract the fractions ($\mathscr{F}$) as shown in Fig. 4, we factor p_A and p_B from the directly measured atom-atom entanglement success probability (P_{ent}). For $d = \{2, 3, 4\}$, the extracted success fractions are $\mathscr{F} = \{0.37(11), 0.64(17), 0.90(24)\}$ respectively, as shown in Fig. 4.

C Magnetic field drift and phase correction

Fig. S2 shows the measured phase evolution over hours of time for each Bell state, with a phase shift of π added manually to all odd parity states $|\Psi^-\rangle$ to bring the curves together. The drifting phase of the coherence for each state in Eq. 3 was fitted to a uniform magnetic field that best reproduced the known ratio of phase shifts given by Table S3.

The rephasing procedure improves fidelity estimates by compensating for slow environmental drifts and ensures accurate characterization of the entangled states. All results shown in the main text include this phase correction. We show the measured populations, contrast and fidelity for each entangled state in Table S5. We also show the detection probabilities for each ion-ion pair at the points maximum contrast in Fig. S4. Note, that the values shown in Figs. S3 and S4 are calculated assuming the Central Limit Theorem, unlike the results shown in Table S5.

D Error budget

Table S4 summarizes the estimated errors contributing to the infidelity of the final entangled states. The total error is separated into two categories: errors common to all entangled states, and those specific to individual Bell states. The common error sources contribute approximately 1.51% to the total infidelity across all measurements. 0.5% of the common error sources are due to SPAM, which is limited by our 1762 nm shelving fidelity to the detection dark state. Our dark state discrimination error is negligible.

State-specific errors vary depending on the magnetic field sensitivity and the number of single-qubit operations required for each of the final Bell states. Decoherence significantly degrades the Bell states that are more sensitive to magnetic field. In addition, we noticed a significant double-excitation error that arises when $m - n = 1$ for photons detected in time-bins m, n. This is caused by infidelity in our 1762 nm shelving pulses during the entanglement generation procedure, as any population leftover in the ground state from the swap pulses may be excited a second time. This error is suppressed for entangled states with $m - n > 1$, because the subsequent swap pulses act as a shelving pulse for the "leftover" population. Note that the final atomic states are different from the heralded time-bin detections because of the swap pulses, specifically the atomic state gets advanced by 1, $n \rightarrow n + 1$. For example, the states $|01\rangle \pm |10\rangle$ are heralded by detections in time bins (0,3).

Bell states with minimal sensitivity to these effects (such as $|01 \pm 10\rangle$ or $|13 \pm 31\rangle$) exhibit fidelities consistent with our prior work on remote entanglement with time-bin photon encoding (*23*). In contrast, states like $|23 \pm 32\rangle$, require additional single qubit rotations and are more prone to decoherence due to higher sensitivities to magnetic field, exhibit significantly higher error rates. The corresponding 1762 nm pulse fidelities for the metastable Zeeman transitions are comparable across all components and are primarily limited by common technical noise sources, such as laser phase noise and intensity fluctuations, rather than by intrinsic differences between the transitions.

Several technical improvements provide a clear pathway toward higher fidelities across the entangled atom-photon and atom-atom states. In particular, enhanced magnetic-field stabilization and passive shielding would extend coherence times well beyond the duration of the entanglement

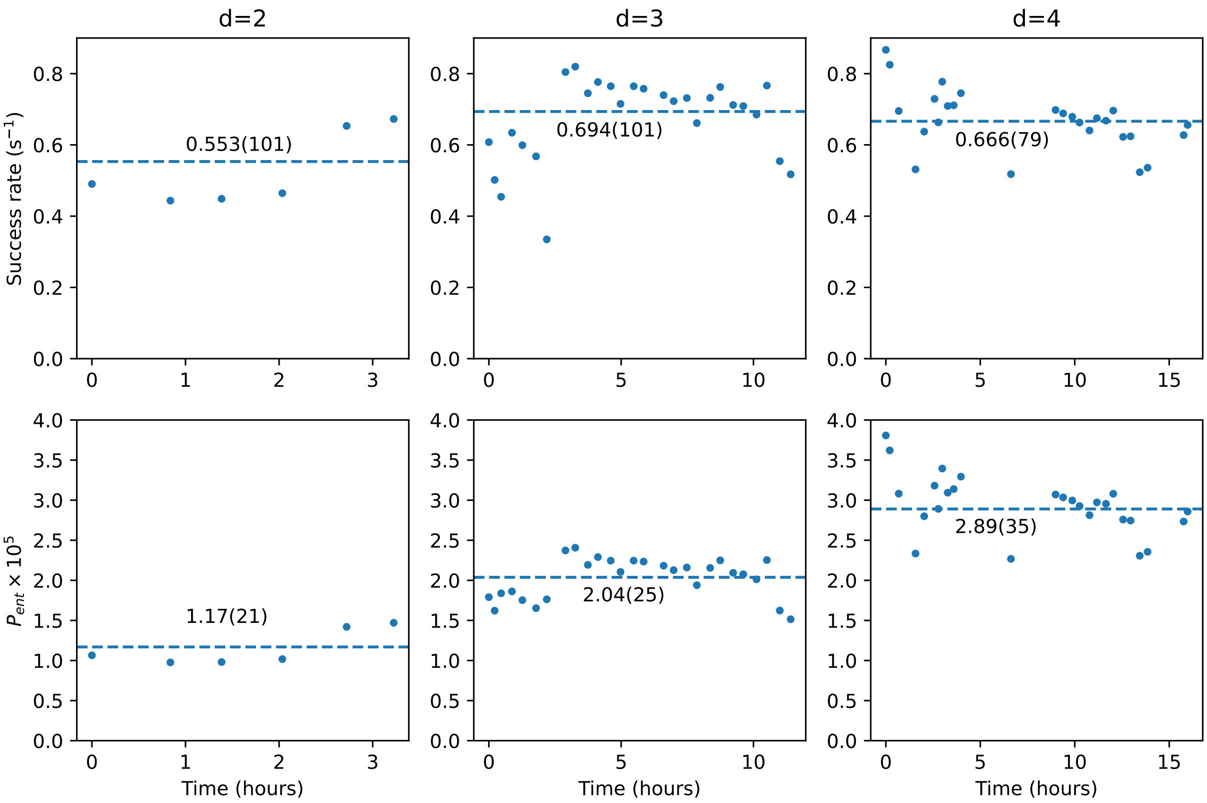

Figure S1: Remote entanglement rates and total entanglement success probabilities for dimensions $d = \{2, 3, 4\}$. Each point represents a separate data set collected over time. Dashed lines indicate the average entanglement rate for each qudit dimension.

sequence, thereby suppressing dephasing (*55*). Further reductions in error can be achieved through faster 1762 nm control pulses (via increased optical power and improved beam focusing), which shortens the protocol duration and proportionally reduces accumulated decoherence. Additional gains are expected from improved phase stabilization of the 1762 nm laser to suppress intensity and phase errors.

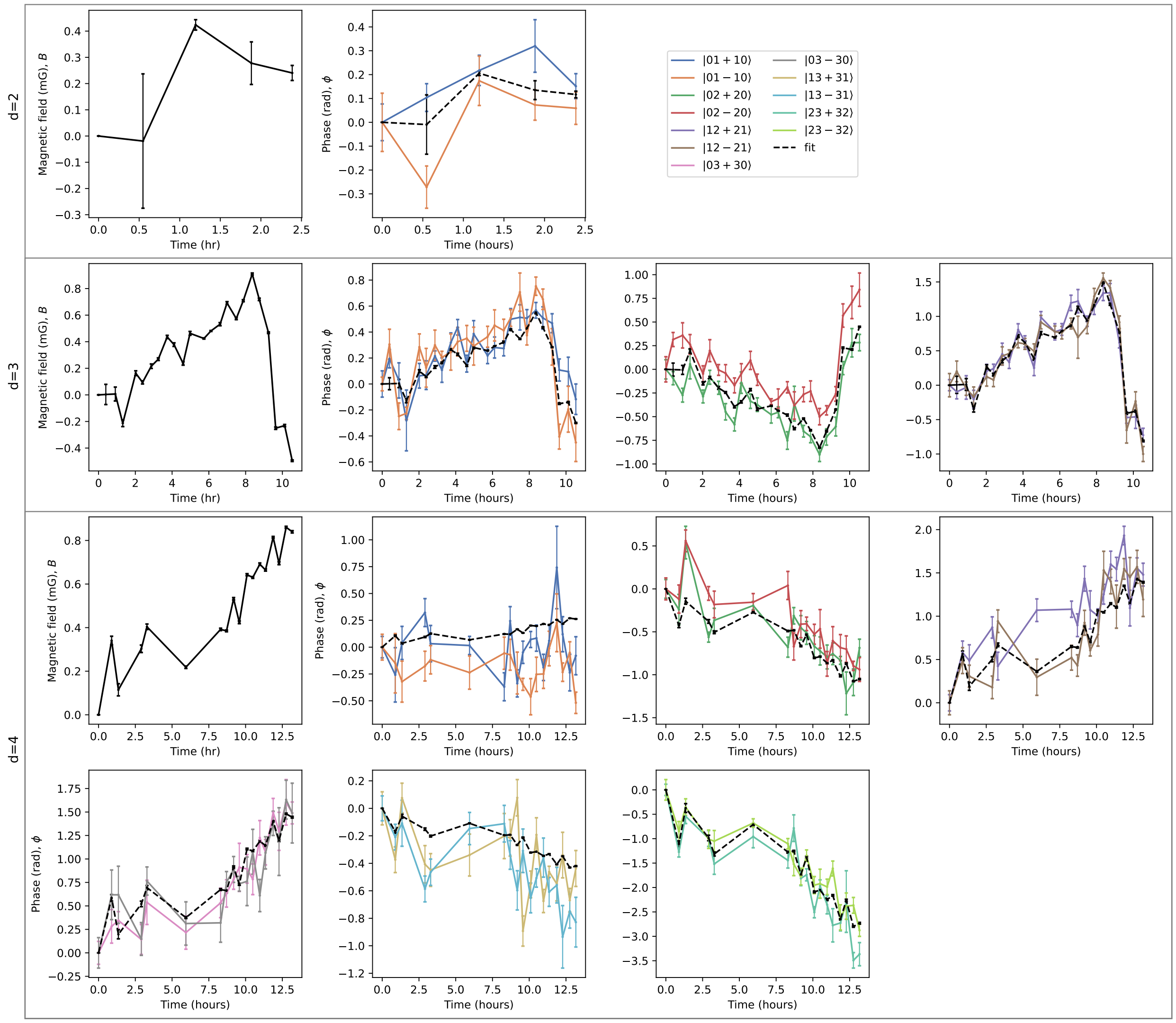

Figure S2: Differential magnetic field drift and phase feed-forward. Solid points are measured phase drifts of all entangled state coherences for qudit dimensions $d = 2, 3, 4$. The dashed lines are fits of the state phases at each point in time to a particular differential magnetic field δB between the two systems that minimizes the deviation from the phase shift $\phi = \delta B \gamma_{nm}(d) T$ using the state sensitivities from Table S3. The fitted differential magnetic field is shown in the first plot of each family of plots.

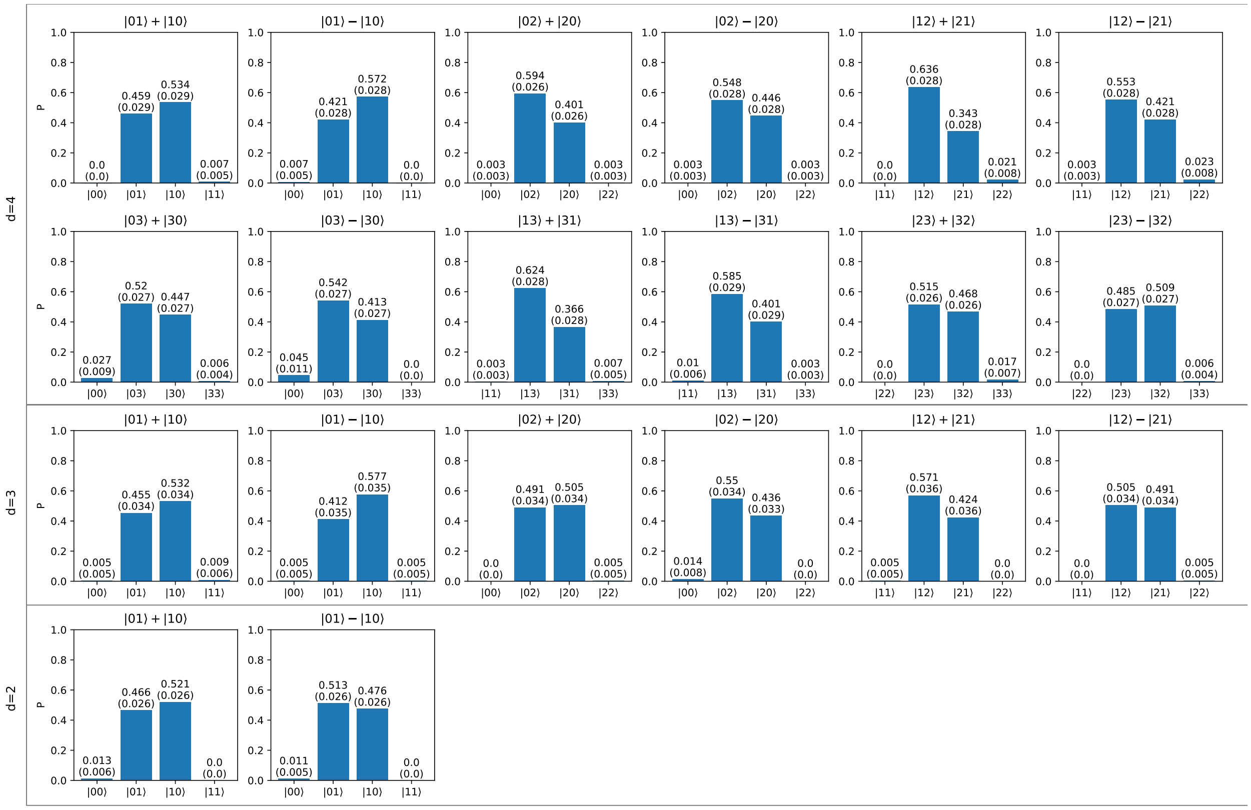

Figure S3: The states of ions A and B after the entanglement heralding.

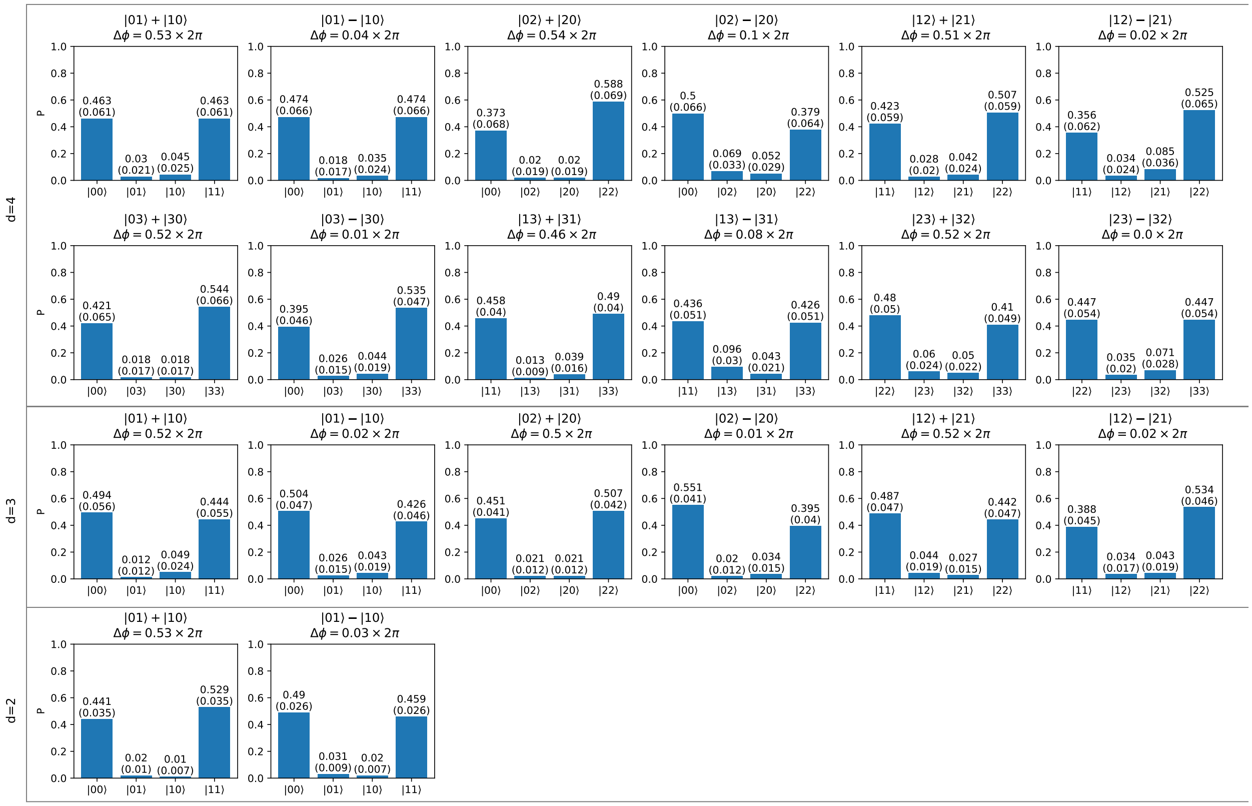

Figure S4: The states of ions A and B after entanglement heralding followed by analysis $\pi/2$-rotation with the phase difference $\Delta\phi$.

d	Attempt length (µs)	Attempt rate (kHz)	Time (s)	Successes	Attempts	Experimental entanglement rate (s^{-1})	P_{ent} ($\times 10^{-5}$)
2	17	58	9,844	5,121	437,972,714	0.553	1.17
3	22.7	44	35,342	23,427	1,149,607,096	0.694	2.04
4	34.1	29	31,315	19,941	689,981,646	0.666	2.89

Table S1: Experimental metrics for different qudit dimensions $d = \{2, 3, 4\}$. Attempt length increases with dimension due to the need for more swaps between levels. Wall-clock time and experimental entanglement rate are calculated with experimental overhead included. The entanglement generation success probability P_{ent} is computed from the number of successes and attempts.

Component	System A	System B
Lens solid angle	0.1	0.2
Fiber coupling	0.30(4)	0.20(3)
Trap clipping	0.78(2)	0.97(1)
Optical losses	0.90(2)	0.80(2)
APD quantum efficiency	0.65(3)	0.65(3)
Excitation probability	0.70(5)	0.70(5)
Branching ratio	0.486	0.486
Total	$p_A = 0.0047(8)$	$p_B = 0.0069(12)$

Table S2: Breakdown of photon collection and detection efficiencies p_A and p_B for individual atom-photon systems A and B.

	Phase sensitivity (rads/mG), γ_{nm}		
Heralded Bell state	d=4	d=3	d=2
$\|10\rangle \pm \|01\rangle$	0.31	0.61	0.49
$\|20\rangle \pm \|02\rangle$	-1.24	-0.92	–
$\|30\rangle \pm \|03\rangle$	1.71	–	–
$\|12\rangle \pm \|21\rangle$	1.64	1.65	–
$\|13\rangle \pm \|31\rangle$	-0.50	–	–
$\|23\rangle \pm \|32\rangle$	-3.22	–	–

Table S3: Bell state phase sensitivities to magnetic field for all heralded states and qudit dimension $d = \{2, 3, 4\}$. These are given by the magnetic g-factors of the relevant states along with dwell times for shelving and swapping operations for each protocol.

Common errors (%)

SPAM	0.5
Photon wavepacket overlap	0.2
Atom recoil, δt = 50 ns	0.3
Background counts	< 0.2
Atom recoil, ω_{qi} fluctuation	< 0.1
Beamsplitter imperfection	< 0.1
Residual erasure errors	< 0.1
Micromotion	< 0.01
**TOTAL common errors** (%)	**1.51**

Errors for individual Bell states (%)

d = 4			
**Bell state**	Decoherence	1762 error	**TOTAL**
$\|01\rangle \pm \|10\rangle$	0.1	0.98	**2.59**
$\|02\rangle \pm \|20\rangle$	0.9	2.61	**5.02**
$\|12\rangle \pm \|21\rangle$	1.6	7.68	**10.79**
$\|03\rangle \pm \|30\rangle$	1.7	7.1	**10.31**
$\|13\rangle \pm \|31\rangle$	0.2	1.19	**2.9**
$\|23\rangle \pm \|32\rangle$	5.9	6.23	**13.64**
d = 3			
**Bell state**	Decoherence	1762 error	**TOTAL**
$\|01\rangle \pm \|10\rangle$	0.2	0.98	**2.69**
$\|02\rangle \pm \|20\rangle$	0.5	10.76	**12.77**
$\|12\rangle \pm \|21\rangle$	1.6	7.12	**10.23**
d = 2			
**Bell state**	Decoherence	1762 error	**TOTAL**
$\|01\rangle \pm \|10\rangle$	0.1	8.6	**10.21**

Table S4: Error budget.

Dimension $d = 2$

State	Fidelity	Populations	Contrast
$\|01\rangle + \|10\rangle$	0.950 (19)	0.986 (6)	0.909 (33)
$\|01\rangle - \|10\rangle$	0.941 (15)	0.988 (5)	0.891 (26)

Dimension $d = 3$

State	Fidelity	Populations	Contrast
$\|01\rangle + \|10\rangle$	0.950 (19)	0.984 (8)	0.909 (33)
$\|01\rangle - \|10\rangle$	0.941 (15)	0.987 (8)	0.891 (26)
$\|02\rangle + \|20\rangle$	0.959 (13)	0.993 (5)	0.925 (23)
$\|02\rangle - \|20\rangle$	0.943 (11)	0.984 (8)	0.893 (17)
$\|12\rangle + \|21\rangle$	0.917 (13)	0.992 (6)	0.857 (17)
$\|12\rangle - \|21\rangle$	0.927 (18)	0.993 (5)	0.881 (26)

Dimension $d = 4$

State	Fidelity	Populations	Contrast
$\|01\rangle + \|10\rangle$	0.950 (19)	0.991 (5)	0.909 (33)
$\|01\rangle - \|10\rangle$	0.941 (15)	0.992 (5)	0.891 (26)
$\|02\rangle + \|20\rangle$	0.959 (13)	0.993 (4)	0.925 (23)
$\|02\rangle - \|20\rangle$	0.943 (11)	0.992 (5)	0.893 (17)
$\|12\rangle + \|21\rangle$	0.917 (13)	0.977 (9)	0.857 (17)
$\|12\rangle - \|21\rangle$	0.927 (18)	0.973 (9)	0.881 (26)
$\|03\rangle + \|30\rangle$	0.901 (18)	0.966 (10)	0.836 (27)
$\|03\rangle - \|30\rangle$	0.864 (18)	0.953 (11)	0.774 (25)
$\|13\rangle + \|31\rangle$	0.987 (13)	0.988 (6)	0.987 (20)
$\|13\rangle - \|31\rangle$	0.971 (12)	0.985 (7)	0.958 (17)
$\|23\rangle + \|32\rangle$	0.862 (18)	0.982 (7)	0.741 (29)
$\|23\rangle - \|32\rangle$	0.849 (23)	0.993 (4)	0.706 (41)

Table S5: Measured fidelities, populations, and contrasts for each entangled Bell state for qudit dimensions $d = \{2, 3, 4\}$.

REFERENCES

1. C. Monroe, J. Kim, Scaling the ion trap quantum processor. *Science* **339**, 1164–1169 (2013).

2. J. P. Covey, H. Weinfurter, H. Bernien, Quantum networks with neutral atom processing nodes. *Npj Quantum Inf.* **9**, 90 (2023).

3. M. Ringbauer, M. Meth, L. Postler, R. Stricker, R. Blatt, P. Schindler, T. Monz, A universal qudit quantum processor with trapped ions. *Nat. Phys.* **18**, 1053–1057 (2022).

4. P. J. Low, B. M. White, A. A. Cox, M. L. Day, C. Senko, Practical trapped-ion protocols for universal qudit-based quantum computing. *Phys. Rev. Res.* **2**, 033128 (2020).

5. P. Jiang Low, B. White, C. Senko, Control and readout of a 13-level trapped ion qudit. arXiv:2306.03340 [quant-ph] (2023).

6. M. Meth, J. Zhang, J. F. Haase, C. Edmunds, L. Postler, A. J. Jena, A. Steiner, L. Dellantonio, R. Blatt, P. Zoller, T. Monz, P. Schindler, C. Muschik, M. Ringbauer, Simulating two-dimensional lattice gauge theories on a qudit quantum computer. *Nat. Phys.* **21**, 570–576 (2025).

7. M. Erhard, M. Krenn, A. Zeilinger, Advances in high-dimensional quantum entanglement. *Nat. Rev. Phys.* **2**, 365–381 (2020).

8. T. Yamazaki, K. Azuma, Linear-optical fusion boosted by high-dimensional entanglement. arXiv:2407.10893 [quant-ph] (2024).

9. S. Ecker, F. Bouchard, L. Bulla, F. Brandt, O. Kohout, F. Steinlechner, R. Fickler, M. Malik, Y. Guryanova, R. Ursin, M. Huber, Overcoming noise in entanglement distribution. *Phys. Rev. X* **9**, 041042 (2019).

10. N. J. Cerf, M. Bourennane, A. Karlsson, N. Gisin, Security of quantum key distribution using d-level systems. *Phys. Rev. Lett.* **88**, 127902 (2002).

11. L. Bulla, M. Pivoluska, K. Hjorth, O. Kohout, J. Lang, S. Ecker, S. P. Neumann, J. Bittermann, R. Kindler, M. Huber, M. Bohmann, R. Ursin, Nonlocal temporal interferometry for highly resilient free-space quantum communication. *Phys. Rev. X* **13**, 021001 (2023).

12. D. Bacco, Y. Ding, K. Dalgaard, X. Cai, X. Zhou, K. Rottwitt, L. K. Oxenløwe, Practical high-dimensional quantum key distribution protocol over deployed multicore fiber. *Nat. Commun* **15**, 45876 (2024).

13. A. Morvan, V. V. Ramasesh, M. S. Blok, J. M. Kreikebaum, K. O'Brien, L. Chen, B. K. Mitchell, R. K. Naik, D. I. Santiago, I. Siddiqi, Qutrit randomized benchmarking. *Phys. Rev. Lett.* **126**, 210504 (2021).

14. M. S. Blok, V. V. Ramasesh, T. Schuster, K. O'Brien, J. M. Kreikebaum, D. Dahlen, A. Morvan, B. Yoshida, N. Y. Yao, I. Siddiqi, Quantum information scrambling on a superconducting qutrit processor. *Phys. Rev. X* **11**, 021010 (2021).

15. M. Mirhosseini, A. Sipahigil, M. Kalaee, O. Painter, Superconducting qubit to optical photon transduction. *Nature* **588**, 599–603 (2020).

16. W. Zhang, D.-S. Ding, M.-X. Dong, S. Shi, K. Wang, S.-L. Liu, Y. Li, Z.-Y. Zhou, B.-S. Shi, G.-C. Guo, Experimental realization of entanglement in multiple degrees of freedom between two quantum memories. *Nat. Commun.* **7**, 13514 (2016).

17. M. Krenn, M. Huber, R. Fickler, R. Lapkiewicz, S. Ramelow, A. Zeilinger, Generation and confirmation of a (100 x 100)-dimensional entangled quantum system. *Proc. Natl. Acad. Sci. U.S.A.* **111**, 6243–6247 (2014).

18. M. Saffman, K. Mølmer, Scaling the neutral-atom rydberg gate quantum computer by collective encoding in holmium atoms. *Phys. Rev. A* **78**, 012336 (2008).

19. Z. Jia, W. Huie, L. Li, W. K. C. Sun, X. Hu, Aakash, H. Kogan, A. Karve, J. Y. Lee, J. P. Covey, An architecture for two-qubit encoding in neutral ytterbium-171 atoms. *Npj Quantum Inf.* **10**, 106 (2024).

20. S. Omanakuttan, A. Mitra, M. J. Martin, I. H. Deutsch, Quantum optimal control of ten-level nuclear spin qudits in ^{87}Sr. *Phys. Rev. A* **104**, L060401 (2021).

21. C. Crocker, M. Lichtman, K. Sosnova, A. Carter, S. Scarano, C. Monroe, High purity single photons entangled with an atomic qubit. *Opt. Express* **27**, 28143–28149 (2019).

22. L. J. Stephenson, D. P. Nadlinger, B. C. Nichol, S. An, P. Drmota, T. G. Ballance, K. Thirumalai, J. F. Goodwin, D. M. Lucas, C. J. Ballance, High-rate, high-fidelity entanglement of qubits across an elementary quantum network. *Phys. Rev. Lett.* **124**, 110501 (2020).

23. S. Saha, M. Shalaev, J. O'Reilly, I. Goetting, G. Toh, A. Kalakuntla, Y. Yu, C. Monroe, High-fidelity remote entanglement of trapped atoms mediated by time-bin photons. *Nat. Commun.* **16**, 2533 (2025).

24. S. Ritter, C. Nölleke, C. Hahn, A. Reiserer, A. Neuzner, M. Uphoff, M. Mücke, E. Figueroa, J. Bochmann, G. Rempe, An elementary quantum network of single atoms in optical cavities. *Nature* **484**, 195–200 (2012).

25. T. van Leent, M. Bock, F. Fertig, R. Garthoff, S. Eppelt, Y. Zhou, P. Malik, M. Seubert, T. Bauer, W. Rosenfeld, W. Zhang, C. Becher, H. Weinfurter, Entangling single atoms over 33km telecom fibre. *Nature* **607**, 69–73 (2022).

26. P. F. Wang, C. Y. Luan, M. Qiao, M. Um, J. H. Zhang, Y. Wang, X. Yuan, M. L. Gu, J. N. Zhang, K. W. Kim, Single ion qubit with estimated coherence time exceeding one hour. *Nat. Commun.* **12**, 233 (2021).

27. A. S. Sotirova, J. D. Leppard, A. Vazquez-Brennan, S. M. Decoppet, F. Pokorny, M. Malinowski, C. J. Ballance, High-fidelity heralded quantum state preparation and measurement. arXiv:2409.05805 [quant-ph] (2024).

28. C. M. Löschnauer, J. Mosca Toba, A. C. Hughes, S. A. King, M. A. Weber, R. Srinivas, R. Matt, R. Nourshargh, D. T. C. Allcock, C. J. Ballance, C. Matthiesen, M. Malinowski, T. P. Harty, Scalable, high-fidelity all-electronic control of trapped-ion qubits. arXiv:2407.07694 [quant-ph] (2024).

29. R. Finkelstein, R. B.-S. Tsai, X. Sun, P. Scholl, S. Direkci, T. Gefen, J. Choi, A. L. Shaw, M. Endres, Universal quantum operations and ancilla-based read-out for tweezer clocks. *Nature* **634**, 321–327 (2024).

30. S. D. Barrett, P. Kok, Efficient high-fidelity quantum computation using matter qubits and linear optics. *Phys. Rev. A* **71**, 060310 (2005).

31. Y. Yu, S. Saha, M. Shalaev, G. Toh, J. OReilly, I. Goetting, A. Kalakuntla, C. Monroe, Entanglement fidelity limits of photonically-networked atomic qubits from recoil and timing. arXiv:2503.19818 [quant-ph] (2025).

32. S. Kikura, R. Inoue, H. Yamasaki, A. Goban, S. Sunami, Taming recoil effect in cavity-assisted quantum interconnects. arXiv:2502.14859 [physics.atom-ph] (2025).

33. J. Apolın, D. P. Nadlinger, Recoil-induced errors and their correction in photon-mediated entanglement between atom qubits. arXiv:2503.16837 [quant-ph] (2025).

34. K. M. R. Audenaert, M. B. Plenio, When are correlations quantum?—Verification and quantification of entanglement by simple measurements. *New J. Phys.* **8**, 266 (2006).

35. F. Bouchard, A. Sit, Y. W. Zhang, R. Fickler, F. M. Miatto, Y. Yao, F. Sciarrino, E. Karimi, Two-photon interference: The hong-ou-mandel effect. *Rep. Prog. Phys.* **84**, 012402 (2021).

36. K. Azuma, S. E. Economou, D. Elkouss, P. Hilaire, L. Jiang, H. K. Lo, I. Tzitrin, Quantum repeaters: From quantum networks to the quantum internet. *Rev. Mod. Phys.* **95**, 045006 (2023).

37. J. O'Reilly, G. Toh, I. Goetting, S. Saha, M. Shalaev, A. L. Carter, A. Risinger, A. Kalakuntla, T. Li, A. Verma, C. Monroe, Fast photon-mediated entanglement of continuously cooled trapped ions for quantum networking. *Phys. Rev. Lett.* **133**, 090802 (2024).

38. C. A. Sackett, D. Kielpinski, B. E. King, C. Langer, V. Meyer, C. J. Myatt, M. Rowe, Q. A. Turchette, W. M. Itano, D. J. Wineland, C. Monroe, Experimental entanglement of four particles. *Nature* **404**, 256–259 (2000).

39. J. Calsamiglia, N. Lütkenhaus, Maximum efficiency of a linear-optical bell-state analyzer. *Appl. Phys. B* **72**, 67–71 (2001).

40. S. Ghosh, G. Kar, A. Roy, A. Sen(De), U. Sen, Distinguishability of bell states. *Phys. Rev. Lett.* **87**, 277902 (2001).

41. W. P. Grice, Arbitrarily complete bell-state measurement using only linear optical elements. *Phys. Rev. A* **84**, 042331 (2011).

42. M. J. Bayerbach, S. E. D'Aurelio, P. van Loock, S. Barz, Bell-state measurement exceeding 50% success probability with linear optics. *Sci. Adv.* **9**, eadf4080 (2023).

43. P. G. Kwiat, H. Weinfurter, Embedded bell-state analysis. *Phys. Rev. A* **58**, R2623–R2626 (1998).

44. C. Schuck, G. Huber, C. Kurtsiefer, H. Weinfurter, Complete deterministic linear optics bell state analysis. *Phys. Rev. Lett.* **96**, 190501 (2006).

45. Y. H. Kim, S. P. Kulik, Y. Shih, Quantum teleportation of a polarization state with a complete bell state measurement. *Phys. Rev. Lett.* **86**, 1370–1373 (2001).

46. E. Knill, R. Laflamme, G. J. Milburn, A scheme for efficient quantum computation with linear optics. *Nature* **409**, 46–52 (2001).

47. H. Aghaee Rad, T. Ainsworth, R. N. Alexander, B. Altieri, M. F. Askarani, R. Baby, L. Banchi, B. Q. Baragiola, J. E. Bourassa, R. S. Chadwick, I. Charania, H. Chen, M. J. Collins, P. Contu, N. D'Arcy, G. Dauphinais, R. De Prins, D. Deschenes, I. Di Luch, S. Duque, P. Edke, S. E. Fayer, S. Ferracin, H. Ferretti, J. Gefaell, S. Glancy, C. González-Arciniegas, T. Grainge, Z. Han, J. Hastrup, L. G. Helt, T. Hillmann, J. Hundal, S. Izumi, T. Jaeken, M. Jonas, S. Kocsis, I. Krasnokutska, M. V. Larsen, P. Laskowski, F. Laudenbach, J. Lavoie, M. Li, E. Lomonte, C. E. Lopetegui, B. Luey, A. P. Lund, C. Ma, L. S. Madsen, D. H. Mahler, L. Mantilla Calderón, M. Menotti, F. M. Miatto, B. Morrison, P. J. Nadkarni, T. Nakamura, L. Neuhaus, Z. Niu, R. Noro, K. Papirov, A. Pesah, D. S. Phillips, W. N. Plick, T. Rogalsky, F. Rortais, J. Sabines-Chesterking, S. Safavi-Bayat, E. Sazhaev, M. Seymour, K. Rezaei Shad, M. Silverman, S. A. Srinivasan, M. Stephan, Q. Y. Tang, J. F. Tasker, Y. S. Teo, R. B. Then, J.

E. Tremblay, I. Tzitrin, V. D. Vaidya, M. Vasmer, Z. Vernon, L. F. S. S. M. Villalobos, B. W. Walshe, R. Weil, X. Xin, X. Yan, Y. Yao, M. Zamani Abnili, Y. Zhang, Scaling and networking a modular photonic quantum computer. *Nature* **638**, 912–919 (2025).

48. J. I. Cirac, P. Zoller, H. J. Kimble, H. Mabuchi, Quantum state transfer and entanglement distribution among distant nodes in a quantum network. *Phys. Rev. Lett.* **78**, 3221–3224 (1997).

49. T. Wilk, S. C. Webster, A. Kuhn, G. Rempe, Single-atom single-photon quantum interface. *Science* **317**, 488–490 (2007).

50. C. Kurz, M. Schug, P. Eich, J. Huwer, P. Muller, J. Eschner, Experimental protocol for high-fidelity heralded photon-to-atom quantum state transfer. *Nat. Commun.* **5**, 5527 (2014).

51. Y. H. Luo, H. S. Zhong, M. Erhard, X. L. Wang, L. C. Peng, M. Krenn, X. Jiang, L. Li, N. L. Liu, C. Y. Lu, A. Zeilinger, J. W. Pan, Quantum teleportation in high dimensions. *Phys. Rev. Lett.* **123**, (2019).

52. P. Hrmo, B. Wilhelm, L. Gerster, M. W. van Mourik, M. Huber, R. Blatt, P. Schindler, T. Monz, M. Ringbauer, Native qudit entanglement in a trapped ion quantum processor. *Nat. Commun.* **14**, 2242 (2023).

53. D. Gottesman, I. L. Chuang, Demonstrating the viability of universal quantum computation using teleportation and single-qubit operations. *Nature* **402**, 390–393 (1999).

54. L. Li, X. Hu, Z. Jia, W. Huie, W. K. Calvin Sun, Aakash, Y. Dong, N. Hiri-O-Tuppa, J. P. Covey, Parallelized telecom quantum networking with a ytterbium-171 atom array. arXiv:2502.17406 [quant-ph] (2025).

55. T. Ruster, C. T. Schmiegelow, H. Kaufmann, C. Warschburger, F. Schmidt-Kaler, U. G. Poschinger, A long-lived zeeman trapped-ion qubit. *Appl. Phys. B* **122**, 254 (2016).
